# MOKCa-3D database: functional and structural analysis of missense mutations in cancer

**DOI:** 10.1093/database/baag001

**Published:** 2026-04-27

**Authors:** Biniam Haile, Adnan Cinar, Sayeda F Banini, Katrin Zhivkova, Finn J Gallagher, Christopher J Richardson, Frances M G Pearl

**Affiliations:** Bioinformatics Laboratory, Faculty of Science, Engineering and Medicine, University of Sussex, John Maynard Smith (JMS) Building, Falmer, Brighton, BN1 9RJ, United Kingdom; Bioinformatics Laboratory, Faculty of Science, Engineering and Medicine, University of Sussex, John Maynard Smith (JMS) Building, Falmer, Brighton, BN1 9RJ, United Kingdom; Bioinformatics Laboratory, Faculty of Science, Engineering and Medicine, University of Sussex, John Maynard Smith (JMS) Building, Falmer, Brighton, BN1 9RJ, United Kingdom; Bioinformatics Laboratory, Faculty of Science, Engineering and Medicine, University of Sussex, John Maynard Smith (JMS) Building, Falmer, Brighton, BN1 9RJ, United Kingdom; Bioinformatics Laboratory, Faculty of Science, Engineering and Medicine, University of Sussex, John Maynard Smith (JMS) Building, Falmer, Brighton, BN1 9RJ, United Kingdom; Division of Structural Biology, Institute of Cancer Research, Chester Beatty Laboratories, 237 Fulham Road, London, SW1E 6BT United Kingdom; Bioinformatics Laboratory, Faculty of Science, Engineering and Medicine, University of Sussex, John Maynard Smith (JMS) Building, Falmer, Brighton, BN1 9RJ, United Kingdom

## Abstract

Determining the functional consequence of missense mutations acquired in the development of cancer is critical to the understanding of the evolution and the therapeutic vulnerabilities of an individual tumour. Several million missense mutations associated with cancer have been reported across different databases with little functional annotation accompanying each mutation. We have designed the MOKCa-3D database, (https://bioinformaticslab.sussex.ac.uk/MOKCa-3D/) to enable the contextualization and interpretation of cancer somatic missense mutations, including the structural impact of the mutation on the 3D structure, and whether the mutation results in a gain or loss of the protein’s function. For each protein, a sequence feature viewer enables interactive visualization of the amino acid sequence, missense mutations, post-translational modification sites, protein domains, active sites, binding sites, protein–protein interaction sites, and mutational frequency. The mutation-level page concisely presents functional insights for each individual mutation, and an interactive MOL* viewer highlights mutated residue on an AlphaFold protein structural model. The SAAP structural impact analysis pipeline was used to identify the structural impact of the mutation. MOKCa-3D concisely presents functional insights and structural impacts of cancer somatic missense mutations enabling users to interpret their functional consequences. It is freely accessible and easy to navigate, making it usable by the widest range of researchers.

## Introduction

Cancer is typically characterized by its uncontrolled cell proliferation, which is caused by the accumulation of mutations in critical sites of genes that are involved in cell cycle regulation, DNA damage repair, and apoptosis [1, 2]. These mutations can be inherited or acquired during the lifetime of an individual due to exposure to environmental hazards like radiation, chemicals, and viral infections [3], as well as inherent cellular processes. For instance, mutations in BRCA1 and BRCA2 genes are associated with an increased risk of developing breast and ovarian cancers. Understanding these mutations has led to targeted cancer therapies [4].

Alterations of the nucleotide sequence of a gene can have a variety of effects on the encoded protein, including frameshift and missense mutations that impact the protein’s structure and function. The most common alteration is a single base change, which usually leads to a missense mutation of a single amino acid or, more rarely, the introduction of a stop codon.

Cancer-associated missense somatic mutations are identified from analysis of tumour DNA sequences. For example, over three million distinct somatic missense mutations have been identified in COSMIC v98 [5]. However, only a small fraction of these somatic missense mutations are likely to be ‘driver’ mutations that contribute to tumorigenesis. It’s important to distinguish those that initiate and drive tumour progression from ‘passenger’ mutations that are not contributing to the cancer phenotype but are the result of the increased mutational rate that occurs in most cancers.

Understanding which mutations are passengers and which are likely to be drivers requires a highly detailed analysis of each mutation in terms of the structural and functional impact it has on the encoded protein, using statistical tools and machine learning methods.

Most existing mutation databases and web tools for annotating and storing cancer-related mutations lack critical insights into the structural impact of mutations. Furthermore, they often fail to determine whether a mutation is a driver and, if so, whether it results in a gain of function (GOF) or loss of function (LOF) of the protein [6, 7]. The MOKCa-3D database presents structural impact and functional annotation of missense mutations in more comprehensive way, including GOF and LOF assessment for known driver mutations curated from the literature.

## Building the MOKCa-3D database

### Mutation data collection and processing

**Figure 1.**
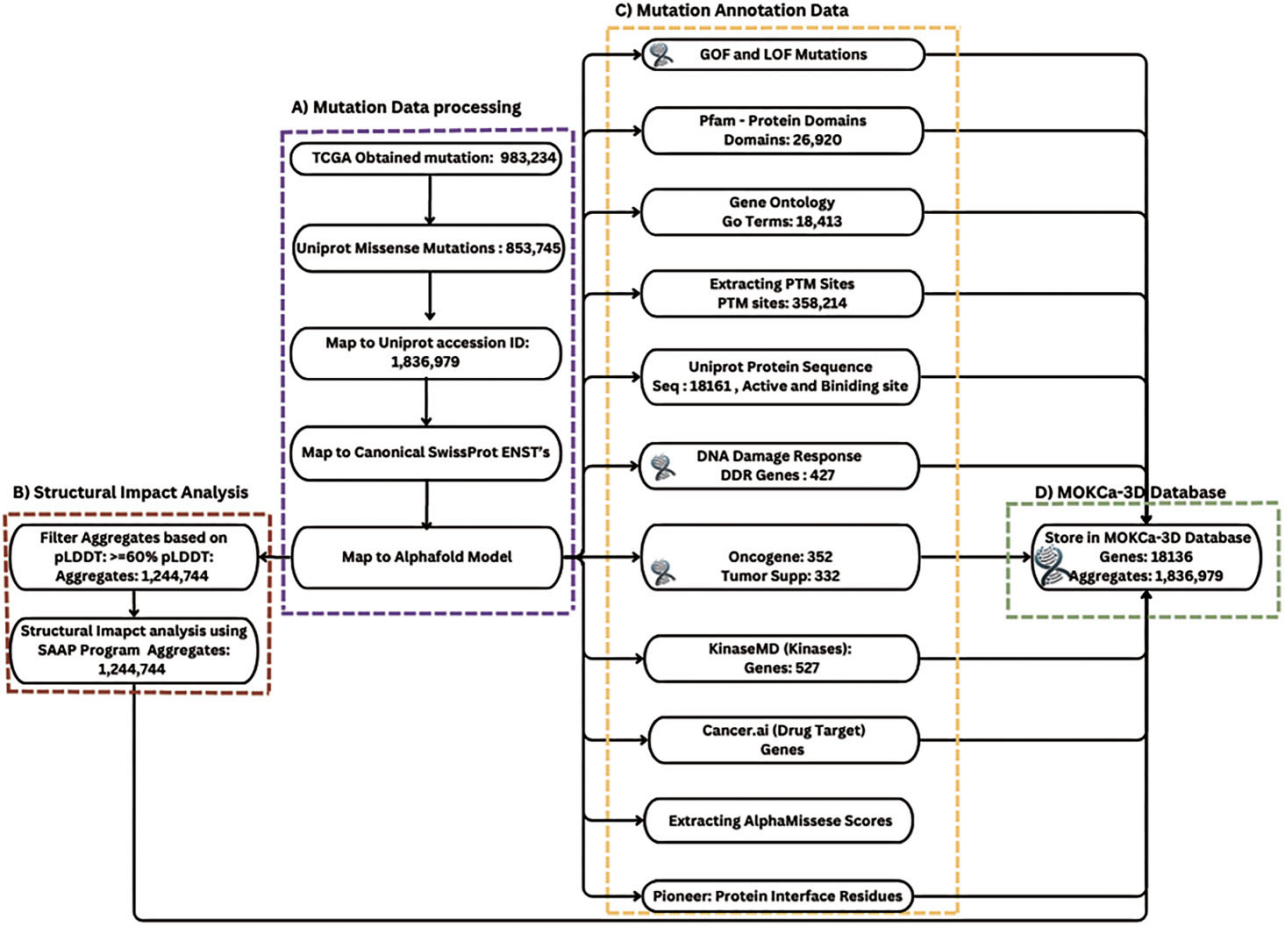
MOKCa-3D data processing workflow. This figure shows the overall workflow consisting of four major components. (A) Mutations data processing—collection, filtering, normalization, and mapping of cancer associated mutations from TCGA and UniProt, (B) structural impact analysis—mapping mutations onto 3D protein structures and assessing those located on AlphaFold models with pLDDT > 60 using SAAP to evaluate structural consequence, (C) mutations annotation data—collecting and processing functional annotation, including GOF and LOF classification from curated literature, post-translational modifications, pfam domains, and protein interface residue, and (D) MySQL Database—all processed mutations data and annotations were stored in relational database for efficient retrieval.

Data collection and processing are crucial to standardizing data and ensuring its quality. The overall data processing and analysis workflow is shown below in Fig. 1. Mutation data were obtained from The Cancer Genome Atlas (TCGA) Research Network using Genomic Data Commons Data Portal (GDC portal) [8] and the UniProt Database [9]. Each mutation was mapped to protein sequences using UniProt accession ID or their Ensembl transcript ID with the biomaRT tool [10]. UniProt Fasta sequences were cross-checked with the mutations to verify wild-type residues [9].

For each sample, if a missense mutation was mapped to multiple transcripts for the same gene, we selected the mutation mapped to the UniProt canonical sequence to be included in MOKCa-3D. When a specific mutation was identified in more than one sample (e.g. BRAF V600E), only one instance of the mutation is recorded, and the frequency of the mutation in MOKCa-3D is calculated. This resulted in 1,836,979 unique missense mutations (aggregates) to include in MOKCa-3D (Fig. 1).

### Annotation data overview

The mutation data processed in this study was enriched with annotations to provide detailed insights into their structural, functional, and pathogenic impacts. MOKCa-3D identifies five gene categories with particular importance in cancer, which are assigned using a variety of online resources. Tumour suppressor genes [5, 7, 11], oncogenes [5, 7, 11], DNA damage response genes, [12], protein kinases (KinaseMD [13]), and current drug targets [7, 14, 15] are all highlighted.

Gene level annotations providing details on the molecular function, biological process, and cellular locations for each protein were retrieved from Amigo2 [16]. For each canonical sequence, residue level annotations were also included to identify whether mutations occurred near or at a functional site. Pfam protein domain assignments were extracted from InterPro [17], and binding sites and active site positions retrieved from UniProt. PTM sites were annotated using PhosphoSitePlus [18], and protein–protein interaction residues were annotated using data from PIONEER [19]. Mutations at or near functional sites have the potential to disrupt protein function by disrupting the regulation of their role in essential biological processes.

For each missense mutation, pathogenicity was assigned using AlphaMissense, which categorizes mutations as pathogenic, benign, or uncertain [20]. Highly recurring mutations were identified. Lastly, gain-of-function and loss-of-function mutations were annotated using assessment curated from literature and from an in-house prediction algorithm (Manuscript in Preparation). These mutations either enhance (GOF) or reduce protein activity (LOF) and can be influential in the progression of cancer and for understanding therapeutic outcomes.

### Structural impact analysis (SAAP)

AlphaFold2 protein structure models of the human proteome were downloaded from EBI [21] and were used to calculate the structural impact of missense mutations using the SAAP structural impact analysis pipeline [22]. To ensure a high level of reliability in these calculations, only mutations where the wild-type residue in the AlphaFold2 model had a pLDDT confidence score of 60% or higher were analysed [21]. Although residues with a pLDDT threshold of 70% or over are seen as reliably modelled, in highly structured regions residues with pLDDTs of 60% can also be reliable (Alessia David, Personal Communication, ISMB 2025). Using a reduced pLDDT score of 60% allowed an extra 85384. mutations to be structurally assessed. This resulted in the structural analysis of 1244744  missense mutations.

The SAAP pipeline checks if a mutation is disruptive to the protein structure by assessing whether a mutation disrupts the native hydrogen bonding in the protein; disrupts a disulfide bond; involves a mutation to a proline residue; is a mutation from a glycine residue; causes a steric clash; introduces a void into the core of the protein; is a mutation to a cis-proline; introduces a charge shift in the core of the protein; introduces a hydrophobic residue on the protein’s surface; or introduces a hydrophilic residue in the protein core [22].

This multi-dimensional annotation strategy provides a comprehensive framework for assessing mutations’ structural and functional effects, offering valuable insights into the consequence of the mutations.

### Database design and backend

**Figure 2.**
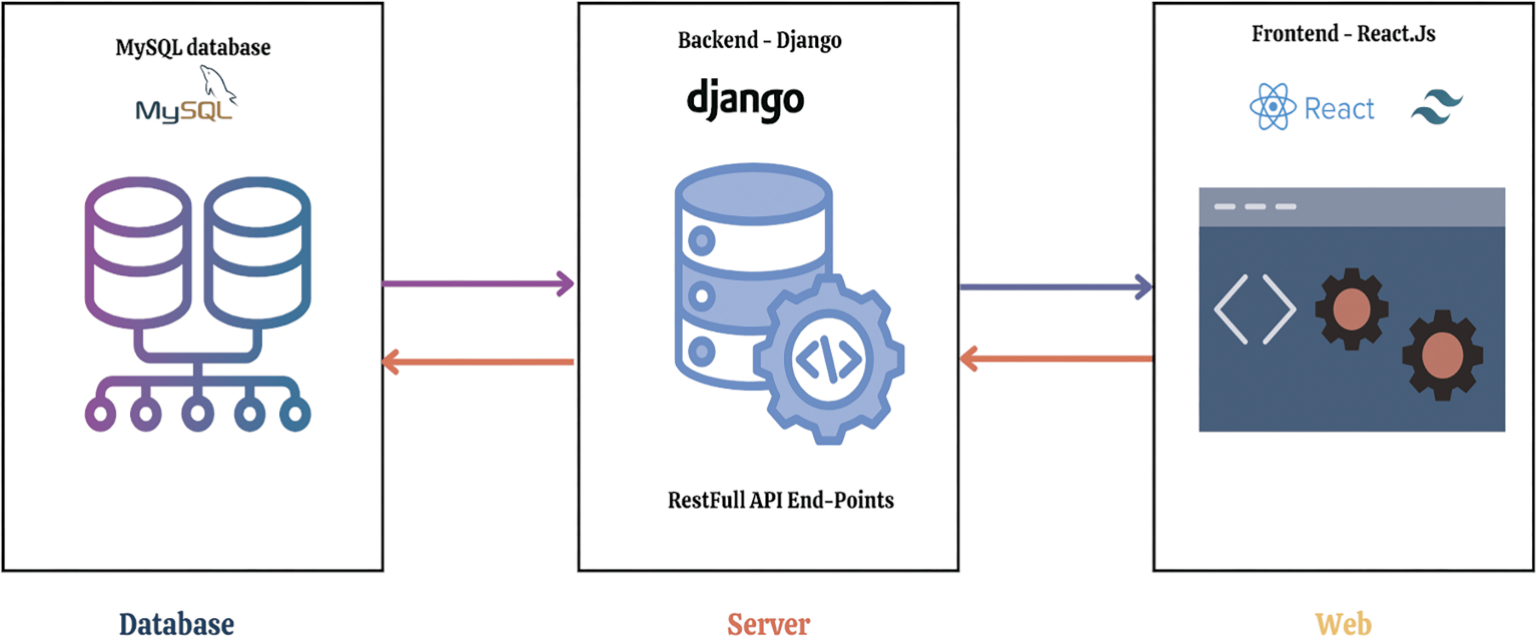
MOKCa-3D overall architectural design. This figure shows the overview of the MOKCa-3D platform architecture, consisting of MySQL relational database for mutation and annotation data, a Django backend for data processing and API endpoints to deliver data, and ReactJS front-end for interactive visualization of structural and functional mutations effect.

MOKCa-3D’s overall architectural design is shown in Fig. 2. All mutation and annotation data are stored in a relational database using MySQL v5.7.24, with a RESTful API backend implemented using Django v5.0.2 with four endpoints to serve data from the database to the frontend via HTTP protocol. This enables scalability, portability, flexibility, and easy integration of the MOKCa-3D server with a web interface that facilitates data presentation, visualization, and interaction. The design was implemented to increase query speed performance, future expansion, and data integrity. Currently, the database schema includes eleven tables (shown in Fig. S3).

## The MOKCa-3D web interface

**Figure 3.**
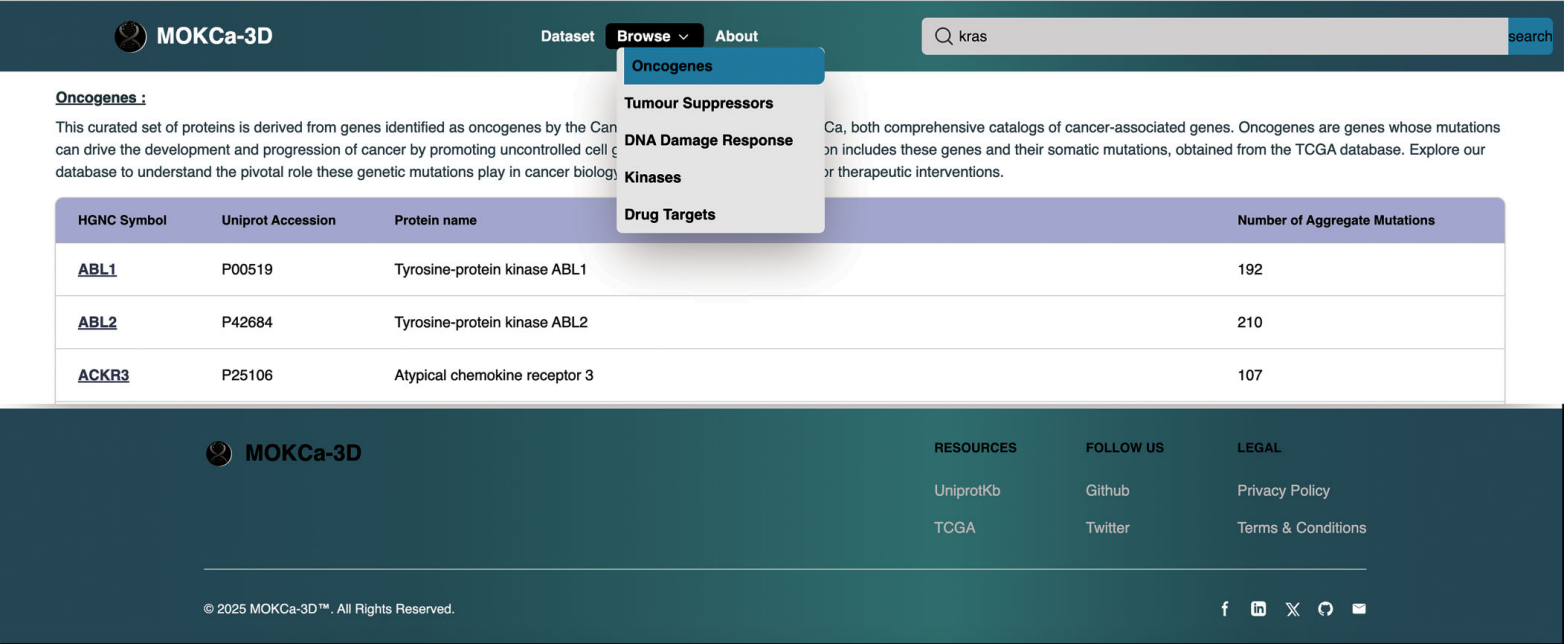
MOKCa-3D web interface. This figure shows user interface for gene-based queries to explore cancer-associated mutations or browse set of genes (Oncogene, Tumour-Suppressor, Protein-kinase, Drug-Target genes, DNA damage response proteins).

The MOKCa-3D web interface (Fig. 3) was implemented using React JS v18.2.0 web framework and Tailwind CSS v3.4.3 for styling. The interface was designed for responsiveness and ease of use. The web interface includes gene level annotation pages and individual mutation level annotations. A Mol* viewer is integrated to visualize protein 3D structure in a more interactive session with mutation residue highlighted [23].

### Gene level annotation

Users can search for specific genes using the HGNC symbol, UniProt accession, or Ensembl ENSG ID, or users can browse the five gene class datasets using the dropdown list. The gene-level page provides gene level information, and links are provided to external databases, including UniProtKB [9], Ensembl [24], CanSar.ai [25], PhosPhositePlus [18], Amigo2 [16], and InterPro [17]. An integrated feature-viewer presents the encoded protein sequence, mutations, PTM-sites, protein interface residues, active sites, binding sites, and protein domain annotation. The interactive graph allows users to inspect individual mutations and see the surrounding functional site annotations. (Fig. S1). Individual mutation pages are accessed from the table below, by clicking on the position of the mutation. This Table also includes the Alpha Missense pathogenicity of the mutational, a GOF/LOF assessment, and the tumour types in which the mutation has been observed. (Fig. S1).

### Mutation level annotation

**Figure 4.**
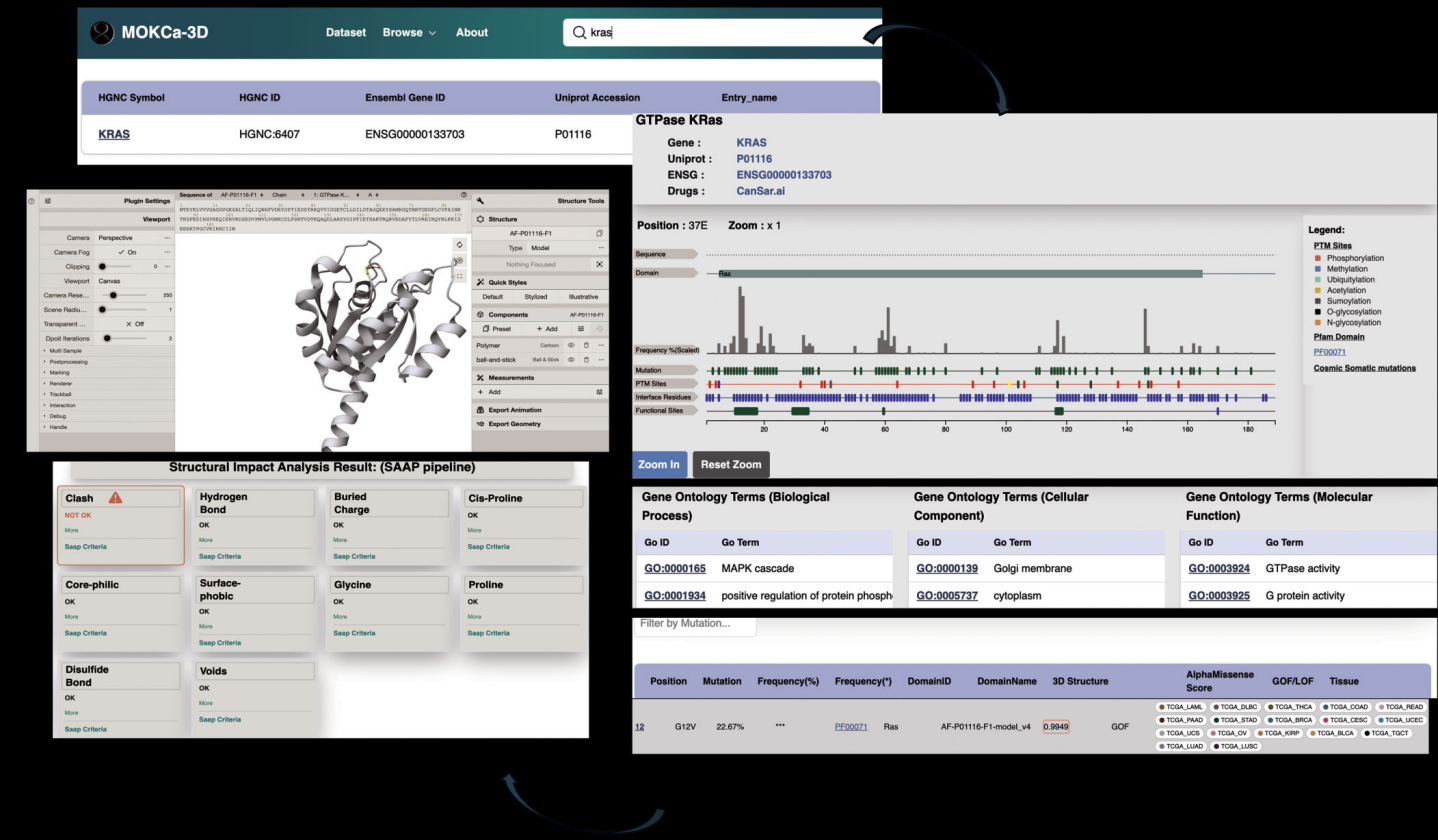
Searching MOKCa-3D. This figure shows an example of use, analysing mutations in KRAS. (A) Gene search page—users begin by querying a gene of interest. (B) Gene-level view (example: KRAS)—displays functional annotations (GO terms) and a filterable mutation list. (C) Mutation-level view (example: G12V)—presents detailed functional and structural annotations along with 3D molecular visualization.

The user can select individual mutations from the gene-level page (Fig. 4). This loads the mutation-level page that provides relevant functional annotation extracted from external databases (Fig. S2). These include the predicted pathogenicity of the mutation and, if available, a GOF/LOF assessment curated from literature with PubMed ID links to the relevant articles. Residue modifications, protein-interface residues, active sites, and binding sites that occur at the position of the mutation or within 3 residues are presented in a list.

An interactive session loads the AlphaFold model and highlights the mutated residue on the structure for further 3D exploration. The structural impact analysis of the mutation is presented in a highly summarized manner, highlighting ten features from SAAP that detail how the mutation affects the structure and potentially impairs protein functionality.

## Example of use

MOKCa-3D allows users to explore specific gene mutation of interest. In this example, we will focus on the *KRAS G12V* mutation, which is associated with various types of tumours, including pancreatic ductal carcinoma, colorectal cancer, and lung adenocarcinoma [26].

To start, users can search for a gene by its name (*KRAS)*, and MOKCa-3D will return it in the search results. By clicking on the gene name, user is directed to the gene-level annotations page. Gene Ontology shows that KRAS is involved in the MAPK cascade, is found in the cytoplasm and the Golgi apparatus, and has GTPase activity. The Pfam link [17] shows that it has a RAS domain, and the links to CanSar.Ai [25] show an assessment of KRAS as a drug target.

The feature viewer [27] displays an interactive visualization showing the protein sequence along with all mutations present in the protein. The mutational frequency shows peaks at positions 12, 13, 117, and 146 in the amino acid sequence. Post-translational modification (PTM) sites, interface residues, functional sites, and protein domains are documented along the length of the protein. This comprehensive visualization allows users to better understand the entire protein and its specific mutations.

In the section below, documented somatic cancer mutations in *KRAS* are displayed in a Table. By clicking on a specific mutation (e.g. G12V) in the table, the user is taken to a detailed mutation page.

The KRAS G12V mutation page shows that this mutation is a documented GOF mutation and provides links to the publications in PubMed. It has a high AlphaMissense pathogenicity score of 0.994. MOKCa-3D further shows that the mutation is located within the Ras domain of the protein, is found in the GTP binding site, and is adjacent to interface residues that are crucial in protein–protein interactions.

The SAAP structural impact analysis indicates that the mutation causes moderate sidechain clashes. This means that the sidechain atoms of the mutated residue clash with surrounding atoms, which could disrupt the protein’s folding and stability. In fact, the KRAS G12V mutation creates a steric block, locking KRAS in its active state and leading to the constitutive activation of its downstream pathways [28].

In the Mol* visualizer, the mutation is highlighted in yellow for further examination (Fig. S2).

## Conclusion and discussion

MOKCa-3D provides comprehensive annotation of somatic missense mutations implicated in different cancer types. The annotations include functional (pathogenicity, GOF, and LOF) and structural (showing how mutation disrupts protein structure) information and empower users to gain a clear understanding of the consequence of the mutation and will help in understanding the molecular mechanism of mutations in tumour growth and progression.

The MOKCa-3D web interface is highly interactive, and responsive and the underlying infrastructure is highly modularized to facilitate future expansion. Planned improvements to the database include the addition of extra annotation data, and the addition of new mutation data.

## Supplementary Material

baag001_Supplemental_Files

## Data Availability

SAAP annotations and GOF/LOF status can be accesed from MOKCa-3D directly. Data derived from a source in the public domain: Missense mutations can be sourced from UniProt: https://www.uniprot.org/. Domain boundaries are derived from InterPro: https://www.ebi.ac.uk/interpro/. PPI data is derived from PIONEER: https://pioneer.yulab.org/. GO terms are derived from: https://geneontology.org/. Data owned by a third party. Tissue type and frequency data is derived from TCGA which can be accessed through the Genomic Data Commons: https://gdc.cancer.gov/. PTMs are derived from Phosphosite: https://www.phosphosite.org/.

## References

[1] Ayob AZ, Ramasamy TS. Cancer stem cells as key drivers of tumour progression. J Biomed Sci. 2018;25:20. 10.1186/s12929-018-0426-4 29506506 PMC5838954

[2] Hanahan D and Weinberg RA. Hallmarks of cancer: the next generation. Cell. 2011;144:646–74. https://10.1016/j.cell.2011.02.013 21376230 10.1016/j.cell.2011.02.013

[3] Malik S, Prasad S, Kishore S et al. A perspective review on impact and molecular mechanism of environmental carcinogens on human health. Biotechnol Genet Eng Rev. 2021;37:178–207. 10.1080/02648725.2021.1991715 34672914

[4] Waarts MR, Stonestrom AJ, Park YC et al. Targeting mutations in cancer. J Clin Invest. 2022;132:e154943. 10.1172/JCI154943 35426374 PMC9012285

[5] Sondka Z, Dhir NB, Carvalho-Silva D et al. COSMIC: a curated database of somatic variants and clinical data for cancer. Nucleic Acids Res. 2024;52:D1210–17. 10.1093/nar/gkad986 38183204 PMC10767972

[6] Baeissa H, Benstead-Hume G, Richardson CJ et al. Identification and analysis of mutational hotspots in oncogenes and tumour suppressors. Oncotarget. 2017;8:21290–304. 10.18632/oncotarget.15514 28423505 PMC5400584

[7] Baeissa HM, Benstead-Hume G, Richardson CJ et al. Mutational patterns in oncogenes and tumour suppressors. Biochem Soc Trans. 2016;44:925–31. 10.1042/BST20160001 27284061

[8] Heath AP, Ferretti V, Agrawal S, et al. The NCI Genomic Data Commons. Nat Genet. 2021;53:257–262. https://10.1038/s41588-021-00791-5 33619384 10.1038/s41588-021-00791-5

[9] UniProt C . UniProt: the universal protein knowledgebase in 2023. Nucleic Acids Res. 2023;51:D523–31. 36408920 10.1093/nar/gkac1052 PMC9825514

[10] Durinck S, Spellman PT, Birney E et al. Mapping identifiers for the integration of genomic datasets with the R/Bioconductor package biomaRt Nat Protoc. 2009;4:1184–91. 10.1038/nprot.2009.97 19617889 PMC3159387

[11] Suehnholz SP, Nissan MH, Zhang H et al. Quantifying the expanding landscape of clinical actionability for patients with cancer. Cancer Discov. 2024;14:49–65. 10.1158/2159-8290.CD-23-0467 37849038 PMC10784742

[12] Pearl LH, Schierz AC, Ward SE et al. Therapeutic opportunities within the DNA damage response. Nat Rev Cancer. 2015;15:166–80. 10.1038/nrc3891 25709118

[13] Hu R, Xu H, Jia P et al. KinaseMD: kinase mutations and drug response database. Nucleic Acids Res. 2021;49:D552–61. 10.1093/nar/gkaa945 33137204 PMC7779064

[14] Knox C, Wilson M, Klinger CM et al. DrugBank 6.0: the DrugBank knowledgebase for 2024. Nucleic Acids Res. 2024;52:D1265–75. 10.1093/nar/gkad976 37953279 PMC10767804

[15] Gingrich PW, Chitsazi R, Biswas A et al. canSAR 2024-an update to the public drug discovery knowledgebase. Nucleic Acids Res. 2025;53:D1287–D1294. https://10.1093/nar/gkae1050 39535036 10.1093/nar/gkae1050 PMC11701553

[16] Carbon S, Ireland A, Mungall CJ et al. AmiGO: online access to ontology and annotation data. Bioinformatics. 2009;25:288–89. 10.1093/bioinformatics/btn615 19033274 PMC2639003

[17] Paysan-Lafosse T, Blum M, Chuguransky S et al. InterPro in 2022. Nucleic Acids Res. 2023;51:D418–27. 10.1093/nar/gkac993 36350672 PMC9825450

[18] Hornbeck PV, Zhang B, Murray B et al. PhosphoSitePlus, 2014: mutations, PTMs and recalibrations. Nucleic Acids Res. 2015;43:D512–20. 10.1093/nar/gku1267 25514926 PMC4383998

[19] Xiong D et al. A structurally informed human protein–protein interactome reveals proteome-wide perturbations caused by disease mutations. Nat Biotechnol. 2024; 43:1510–1524. 39448882 10.1038/s41587-024-02428-4 PMC13399050

[20] Cheng J, Novati G, Pan J et al. Accurate proteome-wide missense variant effect prediction with AlphaMissense. Science. 2023;381:eadg7492. 10.1126/science.adg7492 37733863

[21] Varadi M, Anyango S, Deshpande M et al. AlphaFold protein structure database: massively expanding the structural coverage of protein-sequence space with high-accuracy models. Nucleic Acids Res. 2022;50:D439–44. 10.1093/nar/gkab1061 34791371 PMC8728224

[22] Nouf S, Al-Numair ACM. The SAAP pipeline and database: tools to analyze the impact and predict the pathogenicity of mutations. Bmc Genomics [Electronic Resource]. 2013;14:S4. 10.1186/1471-2164-14-S3-S4 PMC3665582 23819919

[23] Sehnal D, Bittrich S, Deshpande M et al. Mol* viewer: modern web app for 3D visualization and analysis of large biomolecular structures. Nucleic Acids Res. 2021;49:W431–37. 10.1093/nar/gkab314 33956157 PMC8262734

[24] Harrison PW, Amode MR, Austine-Orimoloye O et al. Ensembl 2024. Nucleic Acids Res. 2024;52:D891–99. 10.1093/nar/gkad1049 37953337 PMC10767893

[25] Gingrich PW, Chitsazi R, Biswas A et al. canSAR 2024-an update to the public drug discovery knowledgebase. Nucleic Acids Res. 2025;53:D1287–94. 10.1093/nar/gkae1050 39535036 PMC11701553

[26] Renaud S, Falcoz P-E, Schaëffer M et al. Prognostic value of the KRAS G12V mutation in 841 surgically resected Caucasian lung adenocarcinoma cases. Br J Cancer. 2015;113:1206–15. 10.1038/bjc.2015.327 26372703 PMC4647870

[27] Paladin L, Schaeffer M, Gaudet P et al. The feature-viewer: a visualization tool for positional annotations on a sequence. Bioinformatics. 2020;36:3244–45. 10.1093/bioinformatics/btaa055 31985787

[28] Simanshu DK, Nissley DV, McCormick F. RAS proteins and their regulators in human disease. Cell. 2017;170:17–33. 10.1016/j.cell.2017.06.009. 28666118 PMC5555610

