# Supplementary material for: MOKCa-3D database: functional and structural analysis of missense mutations in cancer
Source: Database (Oxford). 2026 Apr 27;2026:baag001. doi: 10.1093/database/baag001 (PMC13112024; doi:10.1093/database/baag001)
Supplement: baag001_Supplemental_Files [file baag001_supplemental_files.zip › Supplementary Information.docx]

**Supplementary Information**

**Figure S1. Gene-Level Page Annotation**

Gene-level interface displaying summary annotations and a table of individual mutations, including AlphaMissense pathogenicity scores, gain- or loss-of-function classification, and tumour-type information.

**Figure S2. Mutation-Level Page Annotation**

Detailed mutation-level display showing structural and functional annotation, with the selected mutation highlighted in yellow on the 3D protein structure with up to 3 amino acid residues also highlighted in different colours to represent different PTM-site types.

**Figure S3. Database Schema**

Schema illustrating the relational design of the MOKCa-3D database, comprising eleven interconnected tables.
