# Supplementary figures and images for: MOKCa-3D database: functional and structural analysis of missense mutations in cancer
Source: Database (Oxford). 2026 Apr 27;2026:baag001. doi: 10.1093/database/baag001 (PMC13112024; doi:10.1093/database/baag001)

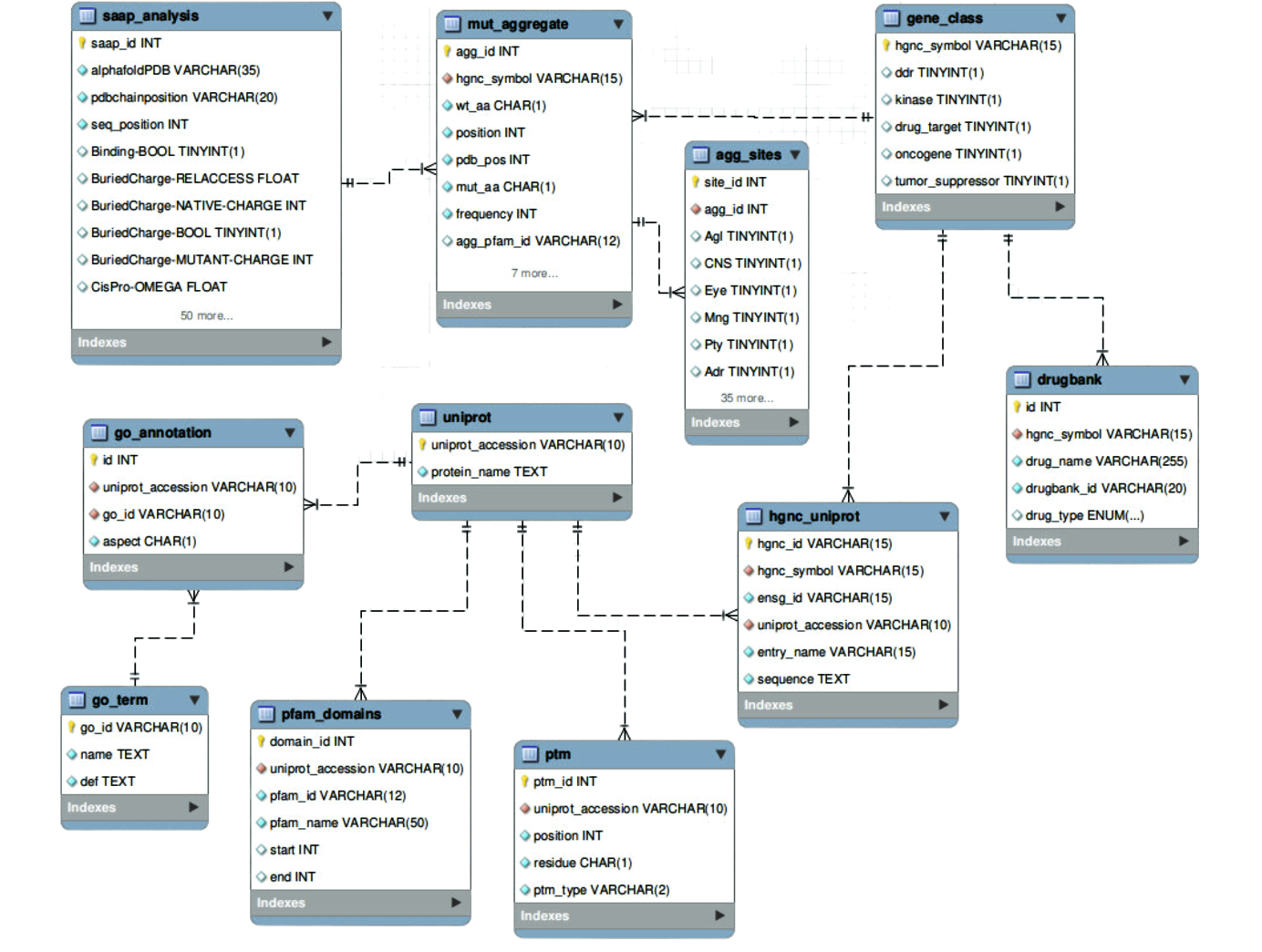

Supplement: baag001_Supplemental_Files [file baag001_supplemental_files.zip › FigureS3_cmykG.tif]
